# Cholesterol-Lowering Effects of Probiotics and Prebiotics: A Review of *in Vivo* and *in Vitro* Findings

**DOI:** 10.3390/ijms11062499

**Published:** 2010-06-17

**Authors:** Lay-Gaik Ooi, Min-Tze Liong

**Affiliations:** School of Industrial Technology, Universiti Sains Malaysia, 11800 Penang, Malaysia; E-Mail: ooilaygaik@yahoo.com

**Keywords:** probiotics, prebiotics, hypocholesterolemic, mechanisms, safety

## Abstract

Probiotics are live microorganisms that promote health benefits upon consumption, while prebiotics are nondigestible food ingredients that selectively stimulate the growth of beneficial microorganisms in the gastrointestinal tract. Probiotics and/or prebiotics could be used as alternative supplements to exert health benefits, including cholesterol-lowering effects on humans. Past *in vivo* studies showed that the administration of probiotics and/or prebiotics are effective in improving lipid profiles, including the reduction of serum/plasma total cholesterol, LDL-cholesterol and triglycerides or increment of HDL-cholesterol. However, other past studies have also shown that probiotics and prebiotics had insignificant effects on lipid profiles, disputing the hypocholesterolemic claim. Additionally, little information is available on the effective dosage of probiotics and prebiotics needed to exert hypocholesterolemic effects. Probiotics and prebiotics have been suggested to reduce cholesterol via various mechanisms. However, more clinical evidence is needed to strengthen these proposals. Safety issues regarding probiotics and/or prebiotics have also been raised despite their long history of safe use. Although probiotic-mediated infections are rare, several cases of systemic infections caused by probiotics have been reported and the issue of antibiotic resistance has sparked much debate. Prebiotics, classified as food ingredients, are generally considered safe, but overconsumption could cause intestinal discomfort. Conscientious prescription of probiotics and/or prebiotics is crucial, especially when administering to specific high risk groups such as infants, the elderly and the immuno-compromised.

## 1. Introduction

The WHO has predicted that by 2030, cardiovascular diseases will remain the leading causes of death, affecting approximately 23.6 million people around the World [1]. It was reported that hypercholesterolemia contributed to 45% of heart attacks in Western Europe and 35% of heart attacks in Central and Eastern Europe from 1999 to 2003 [2]. The risk of heart attack is three times higher in those with hypercholesterolemia, compared to those who have normal blood lipid profiles. The WHO delineated that unhealthy diets such as those high in fat, salt and free sugar, and low in complex carbohydrates, fruits and vegetables, lead to increased risk of cardiovascular diseases [3].

People affected with hypercholesterolemia may avert the use of cholesterol-lowering drugs by practising dietary control or supplementation of probiotics and/or prebiotics. Probiotics are defined as ‘*living microbial supplements that beneficially affect the host animals by improving its intestinal microbial balances*’ [4]. Prebiotics are ‘*indigestible fermented food substrates that selectively stimulate the growth, composition and activity of microflora in gastrointestinal tract and thus improve hosts’ health and well-being*’ [5]. When probiotics and prebiotics are used in combination, they are known as ‘synbiotics’. The use of probiotics and prebiotics has only acquired scientific recognition in recent years although their applications as functional foods have been well-established throughout generations. In the interest of their promising effects on health and well being, probiotics and prebiotics have become increasingly recognized as supplements for human consumption.

In addition to improving gut health, probiotics have also been documented to exert other health-promoting effects such as strengthening of the immune system [6], antihypertensive effects [7], prevention of cancer [8], antioxidative effects [9], reduction of dermatitis symptoms [10], facilitation of mineral absorption [11], amelioration of arthritis [12], reduction of allergic symptoms [13] and improvement of vulvovaginal candidiasis in women [14]. Probiotics have also been studied for their cholesterol-lowering effects [15].

Fructooligosaccharides, inulin, oligofructose, lactulose, and galactooligosaccharides have been identified as prebiotics due to characteristics such as resistance to gastric acidity, hydrolysis by mammalian enzymes and are fermented by gastrointestinal microflora to further selectively stimulate the growth and/or activity of beneficial intestinal bacteria. New compounds with gut resistant properties and selective fermentability by intestinal microorganisms are continuingly being identified and developed as prebiotics [16]. These include oligosaccharides (isomaltooligosaccharides, lactosucrose, xylooligosaccharides and glucooligosaccharides), sugar alcohols and polysaccharides (starch, resistant starch and modified starch) [17]. Generally, prebiotics offer promising health benefits such as improving gastrointestinal microflora by selectively promoting the growth of probiotics and/or inhibition of pathogenic microorganisms [18], stimulation of the immune system [19], cancer prevention [20], stimulation of mineral absorption and bone stability [21] and treatment of irritable bowel-associated diarrhoeas [22]. Prebiotics are utilized by the intestinal microbial population to produce short-chain fatty acids which may lead to the reduced incidence of gastrointestinal disease [23], cancers [24] and cardiovascular diseases [25]; and improvement of lipid profiles [26].

Studies examining the efficacy of probiotics in reducing cholesterol often do not sufficiently address the mechanisms by which probiotics modulate hypocholesterolemic effects and the optimum dose, frequency, and duration of treatment for different probiotic strains. Several mechanisms have been hypothesized, which include enzymatic deconjugation of bile acids by bile-salt hydrolase of probiotics [27], assimilation of cholesterol by probiotics [28], co-precipitation of cholesterol with deconjugated bile [29], cholesterol binding to cell walls of probiotics [30], incorporation of cholesterol into the cellular membranes of probiotics during growth [31], conversion of cholesterol into coprostanol [32] and production of short-chain fatty acids upon fermentation by probiotics in the presence of prebiotics [33].

Probiotics are generally known to be nonpathogenic but they could be infectious, especially in debilitated and immuno-compromised populations [34]. Some species of *Lactobacillus*, *Bifidobacterium*, *Leuconostoc*, *Enterococcus* and *Pediococcus* have been isolated from infection sites [35]. Strains of probiotics have also been found to exhibit antibiotic resistance and have raised concerns about horizontal resistant gene transfer to the host and the pool of gastrointestinal pathogenic microflora [36]. Considering this, the safety verification of probiotics used industrially and commercially is of utmost importance.

## 2. Hypocholesterolemic Potential: *In Vivo* Evidence and Controversies

The use of animals and humans models to evaluate the effects of probiotics and prebiotics on serum cholesterol levels has been emphasized over the years. Human studies have shown promising evidence that well-established probiotics and/or prebiotics possess hypocholesterolemic effects, while new strains of probiotics or new type of prebiotics have been evaluated in animal models for their potential hypocholesterolemic effects. Many studies have used rats [37,38], mice [39], hamsters [40], guinea pigs [41] and pigs [42] as models due to their similarities with humans in terms of cholesterol and bile acid metabolism, plasma lipoprotein distribution, and regulation of hepatic cholesterol enzymes [43]. These animals also share an almost similar digestive anatomy and physiology, nutrient requirements, bioavailability and absorption, and metabolic processes with humans, making them useful experimental models for research applications [42]. Hence, the positive hypocholesterolemic effects shown in animal studies suggest a similar potential in humans. Human trial results that paralleled those obtained from animal studies have further attested to the transferability and reliability of results obtained in selected animal models.

In a study evaluating the effect of *L. plantarum* PH04 (isolated from infant feces) on cholesterol, Nguyen *et al.* [44] administered *L. plantarum* (4 × 10^8^ CFU/mL dose per mouse in daily) to twelve male hypercholesterolemic mice for 14 days. The authors found a significant (*P* < 0.05) reduction of total serum cholesterol (reduced by 7%) and triglycerides (reduced by 10%) compared to the control. In another study, Abd El-Gawad *et al.* [45] conducted a randomized, placebo-controlled and parallel designed study to assess the efficiency of buffalo milk-yogurts (fortified with *Bifidobacterium longum* Bb-46) in exerting a cholesterol-lowering effect. In the study, the authors fed forty-eight male albino hypercholesterolemic rats (average weight 80–100 g) with 50 g of yogurt [contained 0.07% (w/v) *Bifidobacterium longum* Bb-46] daily for 35 days. The administration of *B. longum* Bb-46-fermented buffalo milk-yogurt significantly reduced concentration of total cholesterol by 50.3%, LDL-cholesterol by 56.3% and triglycerides by 51.2% compared to the control (*P* < 0.05). In another study, Fukushima *et al.* [46] found that hypercholesterolemic male Fischer 344/Jcl rats (8 week old) fed with 30 g/kg of *L. acidophilus*-fermented rice bran significantly showed an improved lipid profile compared to the control (without *L. acidophilus*). In this 4-week study, the authors reported a significant (*P* < 0.05) reduction in serum total cholesterol and liver cholesterol of 21.3% and 22.9%, respectively compared to the control. The hypocholesterolemic potential of probiotics has also been evaluated using human subjects. Anderson *et al.* [47] explored the effect of fermented milk containing *L. acidophilus* L1 on serum cholesterol in hypercholesterolemic humans. This randomized, double-blind, placebo-controlled and crossover 10-week study was designed for forty-eight volunteers whose serum cholesterol values ranged from 5.40 mmol/L to 8.32 mmol/L. Daily consumption of 200 g of yogurt containing *L. acidophilus* L1 after each dinner contributed to a significant (*P* < 0.05) reduction in serum cholesterol concentration (−2.4%) compared to the placebo group. In another study, Xiao *et al.* [48] evaluated the effects of a low-fat yogurt containing 10^8^ CFU/g of *B. longum* BL1 on lipid profiles of thirty-two subjects (baseline serum total cholesterol 220–280 mg/dL, body weight 55.4–81.8 kg, aged 28–60 years old). Results from this randomized, single-blind, placebo-controlled and parallel study showed a significant (*P* < 0.05) decline in serum total cholesterol, LDL-cholesterol and triglycerides after 4-weeks. The authors also observed a 14.5% increase in HDL-cholesterol when comparing to the control (yoghurt without *B. longum* BL1; *P* < 0.05).

While the hypocholesterolemic effect of probiotics has been well-documented, prebiotics have also gained increasing attention in cholesterol studies, due to their role in promoting the growth of probiotics. Causey *et al.* [49] conducted a randomized, double-blind and crossover study using hypercholesterolemic subjects to assess the effects of inulin from chicory root on blood cholesterol level. This study involved twelve men that were randomly assigned to two groups, namely the control group (consumed one pint of vanilla ice-cream without inulin) and the inulin group (consumed one pint of vanilla ice-cream containing 20 g of inulin). The 3-week study found that daily intake of 20 g of inulin significantly (*P* < 0.05) reduced serum triglycerides. Similarly, another double-blind, randomized and placebo-controlled crossover study involving eight healthy volunteers with a daily consumption of 10 g of inulin for three weeks has also reached the same conclusion [50]. Plasma triacylglycerol concentrations was significantly (*P* < 0.05) lower compared to the placebo [50]. In another study, Brighenti *et al.* [51] used a randomized, double-blind, placebo-controlled and parallel design trial involving twelve healthy male volunteers to study the effect of prebiotic on lipid profiles. In this 12-week trial, the authors found that the daily consumption of 50 g of a rice-based ready-to-eat cereal containing 18% inulin significantly (*P* < 0.05) reduced plasma total cholesterol and triacylglycerols by 7.9% (±5.4) and 21.2% (±7.8), respectively compared to the control. Similarly, Mortensen *et al.* [52] found that forty male mice fed with a purified diet with 10% of long-chained fructan for 16 weeks showed that the fructan significantly reduced blood cholesterol by 29.7% (*P* < 0.001), LDL-cholesterol concentration by 25.9% (*P* < 0.01), IDL-cholesterol level by 39.4% (*P* < 0.001) and VLDL-cholesterol concentration by 37.3% (*P* < 0.05) compared to the control group.

Other indigestible and fermentable compounds such as germinated barley, oligodextrans, gluconic acid, lactose, glutamine, hemicellulose-rich substrates, resistant starch and its derivatives, lactoferrin-derived peptide, and *N*-acetylchitooligosaccharides [53] have also been identified to exert prebiotic potentials with hypocholesterolemic effects. In a study evaluating the cholesterol-lowering effect of resistant starch, Fernandez *et al.* [43] administered 10 g/100 g of resistant starch (obtained from the Meer Corporation) to male Hartley guinea pigs (body weight of 300–400 g) for four weeks. This randomized, placebo-controlled and parallel designed study used sixteen male guinea pigs and the results showed that the resistant starch significantly reduced (*P* < 0.01) plasma cholesterol by 27.4% and LDL-cholesterol concentration by 28.0% compared to the control group. In another randomized, placebo-controlled and parallel designed study, Wang *et al.* [54] found that ten male hypercholesterolemic Wistar rats (7-week-old; mean body weight of 210 ± 20 g) fed with a starch from Chinese yam (*Dioscorea opposita* cv. Anguo) for eight weeks showed a significantly lower plasma total cholesterol, LDL-cholesterol and triglyceride (*P* < 0.05) than the control (32.8%, 27.5% and 46.2% lower, respectively). Favier *et al.* [55] evaluated the hypocholesterolemic effects of β- cyclodextrin in a randomized, placebo-controlled and parallel design trial involving ten male Wistar rats (mean body weight of 150 g). In this 21-day trial, the authors found that daily consumption of 25 g/kg of β-cyclodextrin significantly (*P* < 0.05) reduced plasma cholesterol and triacylglycerols by 25.9% and 35.0%, respectively, compared to the control group.

Studies have presented evidence of independent hypocholesterolemic effects of probiotics and prebiotics, leading to subsequent evaluations on synbiotics. The administration of a synbiotic product (containing *L. acidophilus* ATCC 4962, fructooligosaccharides, mannitol and inulin) to twenty-four hypercholesterolemic male pigs yielded promising hypocholesterolemic effects [56]. The authors reported a significant reduction of plasma total cholesterol (*P* < 0.001), triacylglycerol (*P* < 0.001) and LDL-cholesterol (*P* < 0.045) in pigs consuming the synbiotic diet for eight weeks compared to the control. Kieβling *et al.* [57] evaluated the hypocholesterolemic effect of a synbiotic yoghurt (containing *L. acidophilus* 145, *B. longum* 913 and oligofructose) in a randomized, placebo-controlled and crossover study involving twenty-nine women. The authors found that the daily consumption of 300 g synbiotic yoghurt over 21 weeks significantly increased (*P* < 0.002) serum HDL-cholesterol by 0.3 mmol/L, leading to an improved ratio of LDL/HDL. In another study, Schaafsma *et al.* [58] conducted a randomized, placebo-controlled, double blind and crossover designed study involving thirty volunteers (aged 33–64 years old; body weight 66.5–98.0 kg) with mean total cholesterol of 5.23 ± 1.03 mmol/L and LDL-cholesterol of 3.42 ± 0.94 mmol/L. In this study, the authors observed that daily consumption of 375 mL synbiotic milk [containing of 10^7^–10^8^ CFU/g of *Lactobacillus acidophilus* and 2.5% (g/100 g) of fructooligosaccharides] resulted in a significant decline in total cholesterol (*P* < 0.001), LDL-cholesterol (*P* < 0.005) and LDL/HDL ratio (*P* < 0.05) of 4.4%, 5.4% and 5.3% respectively.

Although many studies have demonstrated convincing cholesterol-lowering effects of probiotics in both animals and humans, controversial results have surfaced. A study by Hatakka *et al.* [59] refuted the purported hypocholesterolemic effect of probiotics, and reported that the administration of *L. rhamnosus* LC705 (10^10^ CFU/g per capsule; two capsules daily) did not influence blood lipid profiles in thirty-eight men with mean cholesterol levels of 6.2 mmol/L after a 4-week treatment period. In another study involving forty-six volunteers (aged 30–75 years old), Simons *et al.* [60] found that the consumption of *Lactobacillus fermentum,* (2 × 10^9^ CFU per capsule; four capsules daily) did not contribute to any lipid profile changes after 10 weeks. Lewis and Burmeister [61] conducted a randomized, placebo-controlled double blind and crossover designed study to determine the effect of *Lactobacillus acidophilus* on human lipid profiles. In the study, eighty volunteers (aged 20–65 years old; baseline total cholesterol of > 5.0 mmol/L; mean Body Mass Index of 27.8 kg/m^2^) consumed two capsules containing freeze-dried *L. acidophilus* (3 × 10^10^ CFU/2 capsules) three times daily for six weeks, and crossed over for another six weeks after a 6-week washout period. The authors found that *L. acidophilus* capsules did not significantly change plasma total cholesterol, LDL-cholesterol, HDL-cholesterol and triglycerides of the subjects. Similar controversies were also raised from studies evaluating the hypocholesterolemic properties of prebiotics and also when probiotics and prebiotics were used together (synbiotic) (Table 1).

Such controversial findings may be attributed to various factors. Although *in vivo* trials utilize real life models with true representations of the actual pathological systems, these trials are also easily affected by external factors such as different strains of probiotics, varying types of prebiotics, administration dosage, analytical accuracy of lipid analyses, clinical characteristic of subjects, duration of treatment period, inadequate sample sizes, and lack of suitable controls or placebo groups [65,66]. Although some of these studies failed to yield significant results, the reported hypocholesterolemic potential of probiotics and prebiotics supplementation warrants further research.

## 3. Dosage-Response Effects

Although the hypocholesterolemic potential of probiotics and prebiotics has been widely studied, an accurate dosage of administration has yet to be established. There is a lack of dosage-response studies to determine the ‘minimal effective dosage’ of probiotics and/or prebiotics needed to reduce blood cholesterol levels. The concentration of probiotics in food products varies tremendously and there are currently no regulated standards for probiotic products to produce a cholesterol-lowering effect [67]. A review of past studies has revealed that the effective administration dosages of probiotics vary greatly and is dependent on the strains used and the clinical characteristics of subjects, such as lipid profiles. Although probiotics have been delivered in the range of 10^7^ to 10^11^ CFU/day in humans [68] and 10^7^ to 10^9^ CFU/day in animals [69,70], some probiotics have been shown to be efficacious at lower levels, while some require a substantially higher amount to exert a hypocholesterolemic effect.

The administration of *L. plantarum* 299 v at a dosage of 5.0 × 10^7^ CFU/mL daily has been found sufficient to reduce LDL-cholesterol by 12% compared to the control [68]. In contrast, the consumption of probiotic capsules containing *Lactobacillus acidophilus* DDS-1 and *Bifidobacterium longum* (3 × 10^9^ CFU/capsule daily) did not produce significant changes in lipid profiles [71]. This suggests that higher dosage may not necessarily translate to better effects on cholesterol, as compared to lower dosage. Different strains need varying dosage to exhibit hypocholesterolemic effects (Table 2). Clinically effective dosage of probiotics should only be established based on studies of the specific strains conducted in humans.

Similar to probiotics, there is also no recommended daily dosage of prebiotics that specifically exert a hypocholesterolemic effect [74]. Past studies have demonstrated the efficiency of various prebiotics and the combination of prebiotics and oligosaccharides in different dosages. While one study demonstrated the efficacy of lactulose and L-rhamnose in reducing fasting triglycerides, at dosages of 15 g/day and 25 g/day respectively [75], another study showed that arabinogalactan administered in dosages up to 30 g/day produced insignificant effect on lipid profiles [76]. It appears that the hypocholesterolemic effect is specific to the different types of prebiotics (Table 3). These inconsistent findings call for more in-depth studies to ascertain the proper dosage of prebiotics specifically targeting a hypocholesterolemic effect.

Several studies have also been conducted to determine and justify the use of various formulations of synbiotics for cholesterol-lowering effect. Researchers have optimized cholesterol removal by synbiotics in laboratory media. Using response surface methodology (RSM), Liong and Shah [83] demonstrated that *L. casei* ASCC 292 reduced the highest amounts of cholesterol *in vitro* in the presence of fructooligosaccharides (FOS) and maltodextrin. The greatest reduction of cholesterol was observed from the combination of 1.71% (w/v) *L. casei* ASCC 292, 4.95% (w/v) FOS and 6.64% (w/v) maltodextrin. This optimized formulation was further explored in rats [29]. In a 6-week study using twenty-four hypercholesterolemic male Wistar rats, daily oral-administration of the synbiotic product demonstrated promising hypocholesterolemic effects [29]. Rats fed with the synbiotic product (*L. casei* at 10^9^ CFU/g) showed a significantly (*P* < 0.05) lower total cholesterol and triglyceride (16.7% and 27.1% lower, respectively) compared to the control. However, when rats were fed with the diet comprising of *L. casei* and individual FOS or maltodextrin, insignificant difference in lipid profiles was observed. These findings illustrated the different stimulative effects of different prebiotics on probiotics, leading to different hypocholesterolemic outcomes. In another study, Zhang *et al.* [84] optimized the production of a synbiotic for *in vitro* cholesterol removal. The authors also utilized RSM and involved *L. plantarum* LS12 as the probiotic, and galactooligosaccharides (GOS) and mannitol as the prebiotics. The authors observed that *L. plantarum* LS12 in the presence of GOS and mannitol could reduce 75.9% more cholesterol compared to the control (glucose-supplemented medium).

Thus, more studies are needed, not only to determine the effective dosage of synbiotic to exhibit hypocholesterolemic effects, but also to evaluate the effects of symbiosis between probiotics and prebiotics on cholesterol-lowering/removing properties. It is subjective to state a general dosage that could be applied to all probiotic/prebiotic products to exhibit a hypocholesterolemic effect. The prescribed dosage should also be justified by well-designed *in vivo* studies specifically investigating on cholesterol profiles.

## 4. Mechanisms of Cholesterol-Lowering Effects

**Figure 1:**
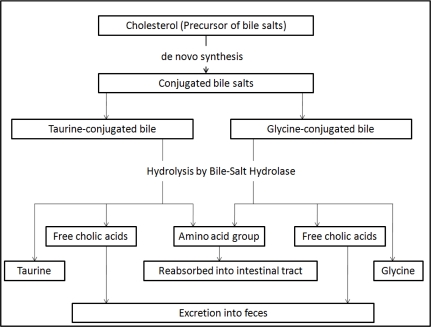
Cholesterol as the precursor for the synthesis of new bile acids and the hypocholesterolemic role of bile salt hydrolase.

Past *in vitro* studies have evaluated a number of mechanisms proposed for the cholesterol-lowering effects of probiotics and prebiotics. One of the purported mechanisms includes enzymatic deconjugation of bile acids by bile-salt hydrolase of probiotics. Bile, a water-soluble end product of cholesterol in the liver, is stored and concentrated in the gallbladder, and released into the duodenum upon ingestion of food [85]. It consists of cholesterol, phospholipids, conjugated bile acids, bile pigments and electrolytes. Once deconjugated, bile acids are less soluble and absorbed by the intestines, leading to their elimination in the feces. Cholesterol is used to synthesize new bile acids in a homeostatic response, resulting in lowering of serum cholesterol [85] (Figure 1). In an *in vitro* study, Jones *et al.* [86] evaluated the role of bile salt hydrolase in cholesterol-lowering using *Lactobacillus plantarum* 80 (pCBH1). Bile salt hydrolase (BSH) is the enzyme responsible for bile salt deconjugation in the enterohepatic circulation. It has been detected in probiotics indigenous to the gastrointestinal tract. The authors found that BSH activity was able to hydrolyze conjugated glycodeoxycholic acid and taurodeoxycholic acid, leading to the deconjugation of glyco- and tauro-bile acids.

**Figure 2:**
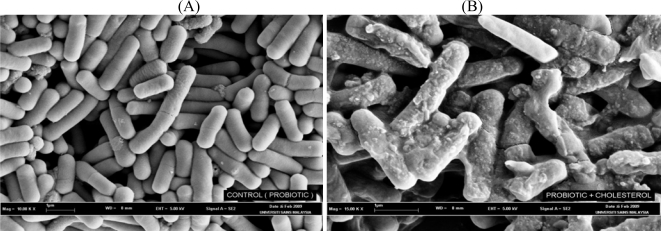
Scanning electron micrograph of *Lactobacillus bulgaricus* cultivated in (A) media without cholesterol and (B) broth supplemented with cholesterol (100 mM).

The hypocholesterolemic effect of the probiotics has also been attributed to their ability to bind cholesterol in the small intestines (Figure 2). Usman and Hosono [87] previously reported that strains of *Lactobacillus gasseri* could remove cholesterol from laboratory media via binding onto cellular surfaces. The ability of cholesterol-binding appeared to be growth and strain specific. Kimoto *et al.* [88] later strengthened such a hypothesis by evaluating the removal of cholesterol by probiotics cells during different growth conditions. Live and growing cells were compared to those that were non-growing (live but suspended in phosphate buffer) and dead (heat-killed). The authors found that although growing cells removed more cholesterol than dead cells, the heat-killed cells could still remove cholesterol from media, indicating that some cholesterol was bound to the cellular surface.

Cholesterol was also removed by probiotics by incorporation into the cellular membranes during growth. Kimoto *et al.* [88] has examined the removal of cholesterol by several strains of lactococci from media. The authors observed a difference in the fatty acid distribution pattern for cells grown in the presence and absence of cholesterol. Lipids of probiotics are predominantly found in the membrane, suggesting that cholesterol incorporated into the cellular membrane had altered the fatty acid composition of the cells. The incorporation of cholesterol into the cellular membrane increased the concentration of saturated and unsaturated fatty acids, leading to increased membrane strength and subsequently higher cellular resistance toward lysis [31]. Lye *et al.* [31] also further evaluated this mechanism by determining the possible locations of the incorporated cholesterol within the membrane phospholipid bilayer of probiotic cells. The authors incorporated fluorescence probes into the membrane bilayer of probiotic cells that were grown in the absence and presence of cholesterol. Enrichment of cholesterol was found in the regions of the phospholipid tails, upper phospholipids, and polar heads of the cellular membrane phospholipid bilayer in cells that were grown in the presence of cholesterol compared to the control cells, indicating incorporation of cholesterol in those regions.

Cholesterol can also be converted in the intestines to coprostanol, which is directly excreted in feces. This decreases the amount of cholesterol being absorbed, leading to a reduced concentration in the physiological cholesterol pool. Possible conversion of cholesterol into coprostanol by bacteria has been evaluated by Chiang *et al.* [89]. In their study, the authors found that cholesterol dehydrogenase/isomerase produced by bacteria such as *Sterolibacterium denitrificans* was responsible for catalyzing the transformation of cholesterol to cholest-4-en-3-one, an intermediate cofactor in the conversion of cholesterol to coprostanol. This served as a fundamental for further evaluations using strains of probiotic bacteria. In a recent *in vitro* study, Lye *et al.* [32] evaluated the conversion of cholesterol to coprostanol by strains of lactobacilli such as *Lactobacillus acidophilus, L. bulgaricus* and *L. casei* ATCC 393 via fluorometric assays. The authors detected both intracellular and extracellular cholesterol reductase in all strains of probiotics examined, indicating possible intracellular and extracellular conversion of cholesterol to coprostanol. The concentration of cholesterol in the medium also decreased upon fermentation by probiotics accompanied by increased concentrations of coprostanol. This mechanism warrants further evaluations as cholesterol reductase is also directly administered to humans to convert cholesterol to coprostanol in the small intestines for a bloodstream cholesterol-lowering effect.

Most of the hypotheses raised to date are based on *in vitro* experiments, and few attempts have been made to evaluate the possible hypocholesterolemic mechanisms based on *in vivo* trials. Most of the *in vivo* trials conducted thus far have focused heavily on verifying the hypocholesterolemic effects of probiotics, rather than the mechanisms involved. Liong *et al.* [56] had evaluated the hypocholesterolemic effect of a synbiotic and the possible mechanisms involved by using 24 crossbred (Large White × Landrace) hypercholesterolemic pigs. In their parallel 8-week study, the authors found that the administration of a synbiotic containing *L. acidophilus* ATCC 4962, fructooligosaccharides, inulin and mannitol decreased plasma total cholesterol, LDL-cholesterol and triacylglycerols compared to the control. These lipoproteins were subsequently subfractionated and characterized. Pigs supplemented with the synbiotic had a lower concentration of cholesteryl esters in the LDL particles, accompanied by a higher concentration of triacylglycerol. Triacylglycerol-enriched LDL particles are more susceptible to hydrolysis and removal from blood, while loss of cholesteryl esters forms smaller and denser LDL particles leading to a higher removal from blood compared to larger LDL particles. The authors also found that the administration of the synbiotic led to higher concentration of cholesteryl esters in the HDL particles. HDL is termed as the beneficial cholesterol attributed to its role of transporting cholesterol to the liver for further hydrolysis. Cholesterol is transported as cholesteryl esters in the core of HDL. Thus, the authors suggested that the synbiotic induced a hypocholesterolemic effect via altering the pathways of cholesteryl esters and lipoprotein transporters.

Prebiotics such as inulin and fructooligosaccharides are soluble, indigestible, viscous and fermentable compounds that contribute to hypocholesterolemia via two mechanisms: decreasing cholesterol absorption accompanied by enhanced cholesterol excretion via feces, and the production of short-chain fatty acids (SCFAs) upon selective fermentation by intestinal bacterial microflora [90]. Using Sprague-Dawley hypercholesterolemic-induced rats (n = 32), Kim and Shin [91] found that the administration of inulin for 4-weeks decreased serum LDL-cholesterol with increased serum HDL-cholesterol levels (*P* < 0.05) compared to the control. Rats fed with inulin also showed higher excretions of fecal lipid and cholesterol compared to the control (*P* < 0.05), mainly attributed to reduced cholesterol absorption. Similar to indigestible fibers, soluble indigestible prebiotics have been postulated to increase the viscosity of the digestive tract and increase the thickness of the unstirred layer in the small intestine, and thus inhibiting the uptake of cholesterol [92]. This may have led to a higher cholesterol catabolism in the liver that contributed to a hypocholesterolemic effect.

Prebiotics are fermented in the colon by large bowel bacteria, yielding short-chain fatty acids (SCFAs) such as butyrate, acetate and propionate. Fermentation of prebiotics involves a variety of metabolic processes in the anaerobic microbial breakdown of organic compounds, yielding energy for microbial growth and the production of SCFAs [79]. Rossi *et al.* [93] found that butyrate was the major fermentation product from inulin, whereas acetate was produced from fructooligosaccharides. The hypocholesterolemic effect of prebiotics has been mainly attributed to SCFAs. Butyrate is known to inhibit liver cholesterol synthesis and provide a source of energy for human colon epithelial cells, meanwhile propionate may inhibit the synthesis of fatty acids in the liver, thereby lowering the rates of triacylglycerol secretion [77]. Propionate is also involved in the control of hepatic cholesterol synthesis and it reduces the rate of cholesterol synthesis which could lead to the lowering of plasma cholesterol levels [77].

In conclusion, the mechanisms proposed for mediating hypocholesterolemic effect by probiotics and/or prebiotics are numerous. Although those hypotheses were proved via *in vitro* studies, the mechanisms are not firmly established and demonstrated in *in vivo* studies. Therefore, more *in vivo* studies are needed to explore the underlying mechanism of cholesterol-lowering effects by probiotics and/or prebiotics in order to have a better understanding of the mechanisms and better formulations for human consumption.

## 5. Safety of Probiotics and Prebiotics

The current use of probiotics does not require approval from the FDA. Although most members of lactobacilli and bifidobacteria are safe for consumption, some species such as *L. rhamnosus*, *L. casei*, *L. paracasei*, *L. leichmannii*, *L. confuses* and *L. plantarum* have been isolated from infectious sites. Lactobacilli are generally known as the normal microflora in the gastrointestinal tract; however, cases such as septicaemia, bacteremia [94–96] and endocarditis [97] have been recorded, inflicting infants, elderly and the immune-compromised [98]. These cases are uncommon and many studies have proven that probiotics were not the contributory mediator and these cases were usually prompted by individual underlying diseases. Some adverse effects might be induced by probiotics, some of which have been documented in the literature, including systemic infections (Table 4), deleterious metabolic activities, possible genetic interactions between probiotics and intestinal microbes and occurrence of antibiotic resistance in probiotics. These cases of adverse effects were often accompanied by other disease, occurrence of gastrointestinal inflammation and lesions, or by an impaired immune system.

The deleterious metabolic activities of probiotics such as mucin degradation and translocation contribute to possible adverse effects upon the consumption. Following the ingestion of probiotics in the gastrointestinal tract, the load of microorganisms passing through the small bowel is increased, which could cause gastrointestinal disturbances including intestinal inflammation. It has been hypothesized that the accumulation of probiotics along the gastrointestinal tract might lead to the risk of intestinal mucus degradation. However, current findings do not support such a suggestion. Ruseler-van Embden *et al.* [99] determined the mucus glycoprotein degradation ability of *Lactobacillus casei* strain GG, *Lactobacillus acidophilus*, *Bifidobacterium bifidum* and a mesophylic lactic culture (isolated from a commercial fermented product) in germ-free rats. The authors reported that the probiotics strains tested did not degrade intestinal mucus glycoproteins and no damage was observed on the intestinal mucus layer. Recently, Abe *et al.* [100] evaluated *Bifidobacterium longum* BB536, *Bifidobacterium breve* M-16V and *Bifidobacterium infantis* M-63 on mucin degradation activity *in vitro*. No mucin degradation activity was observed and the authors further explored the translocation ability of *Bifidobacterium longum* BB536 in ten 4-weeks-old mice. No translocation was detected and no damage was observed on epithelial cells or to the mucosal layer in the ileum, cecum and colon.

The production of bile salt hydrolase (BSH) by probiotic strains could increase the accumulation of deconjugated bile which could be subsequently transformed into detrimental secondary bile acids by intestinal microflora. It has been suggested that the accumulation of potentially cytotoxic secondary bile acids in the enterohepatic circulation could increase the risk of gastrointestinal diseases such as cholestasis and colorectal cancer [101]. Little information is available on such a possibility and to our knowledge, there has been no study that specifically evaluates the detrimental effects of BSH from probiotics on humans. More studies are needed to ascertain that the deleterious effects of BSH from probiotics do not outweigh its benefits.

Adverse immunological effects have been postulated to occur as a result of administration routes, which included oral or parental administration. When probiotics are administered parentally, their cell walls containing peptidoglycan polysaccharides has been postulated to cause adverse effects such as fever, arthritis, cardioangitis, hepatobiliary lesions or autoimmune diseases [102]. Although it has been noted that these effects are mediated by different cytokines, the detailed mechanisms involved have not been substantially understood. Such a claim remains a hypothesis as there is no direct evidence supported by *in vivo* trials, and warrants further investigation.

The adverse effect in terms of genetic interactions between ingested probiotics and the native intestinal microbes has also been a topic of interest. The phenomenon whereby genetic materials represented by plasmids are transferred between microorganisms in our body [103] raises the question of whether genetic exchange may occur between probiotics and the microorganisms in the gastrointestinal tract. Transduction, conjugation and transformation have been identified as the three basic forms of microbial genetic exchange within gut microbial communities [104–106]. It has been suggested that the transformation of intestinal microflora by DNA may be enhanced upon ingestion of bacteria, leading to genetic rearrangements in the pool of gastrointestinal microflora. The gastrointestinal tract is populated by a complex microflora colony. It could act as a pool for the transmission of antibiotic-resistance genes among beneficial bacteria and harmful pathogens. This transmission can consequently lead to the evolution of antibiotic-resistant probiotics [107] and potential emergence of resistant pathogens.

D’Aimmo *et al.* [108] isolated 34 strains of *Lactobacillus* and *Bifidobacterium* and 21 strains of starter culture bacteria (such as *Streptococcus thermophilus*) from dairy products. The authors found that all strains tested were resistant to the antibiotics such as aztreonam, cycloserin, kanamycin, nalidixic acid, polymyxin B and spectinomycin. In another *in vitro* study, Hummel *et al.* [109] evaluated antibiotic resistant genes in 45 strains of probiotics from the genera of *Lactobacillus*, *Streptococcus*, *Lactococcus*, *Pediococcus* and *Leuconostoc* by using polymerase chain reaction (PCR). The authors found that 77.8 % of the probiotic strains examined were resistant to gentamicin, streptomycin and ciprofloxacin. A previous case study also reported such an occurrence in infants. A 6-week-old infant was hospitalized for a scheduled repair of a double-outlet right ventricle and pulmonic stenosis, and had received a broad-spectrum of antibiotics including vancomycin and ceftriaxone. A probiotic supplement containing *Lactobacillus rhamnosus* GG was also introduced through the gastrostomy tube (10 × 10^9^ cells/capsule; one capsule daily). The infant later developed onset fever and marked leukocytosis, and thus blood samples were drawn for culture examination. The blood culture isolates were positive for *Lactobacillus* species, which were resistant to vancomycin, meropenem, ceftriaxone and cefuroxime [35].

Prebiotics such as inulin and oligofructose are present in our daily diet intake. Daily intake of inulin and oligofructose has been estimated at up to 10 g in the population of the United States [110]. The safety of inulin and oligofuctose for food application was evaluated by many legal authorities worldwide, and *in vivo* experimental evidence has not demonstrated any toxic effects [74]. However, prebiotics at very high doses might increase incidences of bloating, flatulence and high osmotic pressure which lead to gastrointestinal discomfort [111]. The effects might vary widely between individuals and depend on the type of food in which the prebiotics are incorporated.

Prebiotics such as fructooligosaccharides (FOS) stimulate the growth of intestinal microflora and increasing organic acid concentrations within the lower part of gastrointestinal tract [79,93] which further contribute to health benefits such as cholesterol-lowering effect and enhancing the resistance to intestinal pathogens. However, some studies found that a high dose of FOS exhibited adverse effects in animal models. In a randomized, placebo-controlled and parallel designed study, Bruggencate *et al*. [112] administered 60 g/kg of FOS into ten specific pathogen–free 8-week-old male Wistar rats (with a mean body weight of 226 g) for 14 days. The rats were also infected with salmonella. FOS increased translocation of salmonella to extraintestinal sites, mucin excretion and cytotoxicity of fecal water in the FOS group compared to the control. FOS was found to impair the intestinal barrier in rats, as indicated by higher intestinal permeability. The authors further extrapolated their findings using humans as a model. In the double-blind, placebo-controlled, crossover designed study with a washout period of two weeks, Bruggencate *et al.* [113] studied the adverse effects of FOS in thirty-four men (aged 18–55 years old). In this study, the subjects consumed either lemonade with 20 g of FOS (purity 93%, Raftilose P95, Orafti) or 6 g of sucrose (placebo) daily. The authors discovered that the consumption of FOS increased flatulence, intestinal bloating and fecal mucin excretion, indicating the occurrence of mucosal irritation. However, the authors found that FOS did not affect the cytotoxicity of fecal water and intestinal permeability, and the overall effects were more moderate than those in rats. It appears that high dosage of FOS can cause minor disturbances in the gastrointestinal tract, but such cases are far from being a risk to life [22].

On the whole, probiotics and prebiotics are safe for consumption due to their low ability of triggering adverse effects [5,67]. At this point, there is no standard analysis or assay suggested for safety assessment on probiotics and prebiotics [67]. However, each probiotic and prebiotic should be evaluated for safety, so that these strains can be isolated for specific purposes at specific dosages to prevent potential adverse reactions.

## 6. Conclusions

Probiotic and/or prebiotics have been widely assessed for their effects on lipid profiles such as total cholesterol, LDL-cholesterol, HDL-cholesterol and triglycerides. However, not all trials have yielded conclusive results. Certain strains of probiotic and types of prebiotic have demonstrated cholesterol-lowering property while others did not. In order to justify the varying cholesterol-lowering effect exhibited by various strains of probiotics or types of prebiotics, researchers have endeavored to reveal the mechanisms of probiotics and/or prebiotics on hypocholesterolemic effect through *in vitro* and *in vivo* studies. Many of the proposed mechanisms and experimental evidence specifically targeting cholesterol-lowering effects remain controversial. Thus, more properly-designed *in vivo* trials may disclose additional understanding and knowledge to eliminate the controversies, to better understand the underlying mechanisms and for better safety assessment prior to consumption.

## Figures and Tables

**Table 1:** Controversial hypocholesterolemic effects of prebiotic and synbiotic.

**Compound(s)**	**Experimental design**	**Subjects**	**Dose; duration of the study**	**Effects**	**Ref.**
**Inulin**	Randomized, placebo-controlled, double-blind & crossover.	8 volunteers.	3–4 g/100 of inulin & wheat fiber daily for 12 weeks.	No significant improvement in lipid profiles.	[62]
**Fructooligo-saccharides (FOS)**	Randomized, placebo-controlled, double-blind & crossover.	10 diabetic patients (6 men and 4 women); with plasma TC of 4.85–5.58 mmol/L.	20 g FOS/day for 4 weeks.	No significant improvement in lipid profiles.	[63]
**Inulin**	Randomized, placebo-controlled, double-blind & crossover designed study; with 2 six-week treatment periods, separated by a six-week washout period.	25 subjects; with baseline LDL-C ranging from 3.36–5.17 mmol/L.	45 g chocolate bar (containing of 18 g of inulin) daily during treatment period.	No significant improvement in lipid profiles.	[64]
***L. acidophilus*&*B. longum*& fructooligo-saccharides (FOS)**	Randomized, single-blind, placebo-controlled & parallel.	55 normocholesterolemic volunteers.	3 capsules of synbiotics product (consisted of 10^9^ CFU/g of *L. acidophilus* & *B. longum*, & 10–15 mg of FOS) once daily for 2 months.	No significant improvement in lipid profiles.	[65]

TC: Total cholesterol; LDL-C: Low-density lipoprotein cholesterol.

**Table 2:** Dosage-response effects of different probiotic strains on lipid profiles.

**Products/Probiotic strains**	**Experimenta l design**	**Animals/Subjects**	**Dose; duration of the study**	**Effects**	**Ref.**
**Animal models**					
*L. plantarum* CK 102(healthy human isolate)	Randomized, placebo-controlled, parallel.	32 Sprague-Dawley (SD) male rats; 5 weeks old; induced hypercholesterolemic; mean BW of 129 ± 1 g.	5.0 × 10^7^ CFU/mL daily, 6 weeks.	TC: 27.9% decrease (*P* < 0.05) LDL-C: 28.7% decrease (*P* < 0.05) TG: 61.6% decrease (*P* < 0.05)	[70]

*L. acidophilus* (wild chickens & human isolates)	Randomized, placebo-controlled, parallel.	30 Awassi weaning lambs; hypercholesterolemic ; mean BW of 55.1 ± 3.4 & 57.9 ± 4.7 kg for the treated & control groups, respectively.	1 × 10^9^ CFU/capsule, 2 capsules daily, 120 days.	TC: 22.6% decrease (*P* < 0.05) [treatment group with mean plasma TC of 72.8 ± 5.7 mg/100 mL; control group with mean plasma TC of 94.0 ± 7.8 mg/100 mL]	[69]
	
*L. plantarum* KCTC3928 (Cellbiotech Co. Ltd, Korea)	Randomized, placebo-controlled, parallel.	21 six-week-old C57BL/6 male mice; induced hypercholesterolemic.	1 × 10^9^ CFU/mL of *L. plantarum* KCTC3928, 4 weeks.	TC: 33% decrease (*P* < 0.05) LDL-C: 42% decrease (*P* < 0.05) TG: 32% decrease (*P* < 0.05) HDL-C: 35% increase (*P* < 0.05)	[72]

**Human models**					
*L. plantarum* 299v (ProViva)	Randomized, placebo-controlled, double-blind, parallel.	36 healthy volunteers with moderately elevated fibrinogen concentrations (>3.0 g/L); 35–45 years old; mean TC of 5.59 ± 0.88 mmol/L for treatment group & 5.51 ± 0.75 mmol/L for control group.	400 mL of rose-hip drink containing 5.0 × 10^7^ CFU/mL daily, 6 weeks.	TC: 2.5% decrease LDL-C: 7.9% decrease	[68]
*Enterococcus faecium* & 2 strains of *Streptococcus thermophilus* (Causido®; Gaio®)	Randomized, placebo controlled, double-blind, crossover.	32 patients; 36–65 years old; mean TC of 248.47 ± 26.75 mg/dL, mean LDL-C of 172.22 ± 21.17 mg/dL.	200 g of Gaio® containing 10^5^–10^9^/mL of *E. faecium* & 5–20 × 10^8^/mL of *S. thermophilus* daily, 16 weeks.	TC: 5.3% decrease (*P* = 0.004) LDL-C: 6.15% decrease (*P* = 0.012)	[73]

TC: Total cholesterol; LDL-C: Low-density lipoprotein cholesterol; HDL-C: High-density lipoprotein cholesterol; TG: triglycerides; BW: body weight.

**Table 3:** Dosage-response effects of different prebiotics/oligosaccharides on lipid profiles.

**Prebiotics/Oligosaccharides**	**Experimental design**	**Animals/Subjects**	**Dose; duration of the study**	**Effects**	**Ref.**
**Animal Models**					
Inulin	Randomized, placebo-controlled, parallel.	10 male golden Syrian hamsters, mean BW of 58 ± 4 g.	16% of inulin daily, 5 weeks.	TC: 29% decrease (*P* < 0.05) TG: 63% decrease (*P* < 0.05)	[77]
Chito-oligosaccharides (COS)	Randomized, placebo-controlled, parallel.	49 male Arbor Acres broiler chickens; 196 days old.	100 mg/kg BW daily, 42 days.	TG: 26.9% decrease (*P* < 0.05) HDL-C: 12.3% increase (*P* < 0.05)	[78]
Xylo-oligosaccharides (XOS)	Randomized, placebo-controlled, parallel.	40 male Sprague-Dawley rats; 6 weeks old.	60 g XOS/kg diet, 35 days.	TG: 33.9% decrease (*P* < 0.05)	[79]
Soybean oligosaccharides	Randomized, placebo-controlled, parallel.	50 Wistar rats; aged of 4-week; induced hypercholesterolemic.	450 mg/kg BW/day, 45 days.	TC: 38.5% decrease (*P* < 0.05) LDL-C: 43.0% decrease (*P* < 0.05) TG: 40.8% decrease (*P* < 0.05) HDL-C: 81.9% increase (*P* < 0.05) (compared to the positive control group)	[80]

**Human Models**					
Inulin	Randomized, placebo-controlled, double-blind, crossover.	8 healthy volunteers; 23–32 years old, BMI of 19–25 kgm^−2^.	10 g/day, 3 weeks.	TG: 16.3% decrease (*P* < 0.05)	[50]
Fructo-oligosaccharides (FOS)	Randomized, placebo-controlled, single-blind, crossover.	20 diabetic & hypercholesterolemic volunteers with fasting serum TC concentrations > 6 mmol/L.	15 g/day, two 20 days treatment period, no washout period between treatments.	HDL-C: 2.8% increase	[81]
Galacto-oligosaccharides	Randomized, placebo-controlled, double-blind, crossover.	44 elderly volunteers (16 men & 28 women); 64–79 years old.	5.5 g/d, two 10 weeks treatment period, 4-week washout period.	No significant improvement in lipid profiles.	[82]

TC: Total cholesterol; LDL-C: Low-density lipoprotein cholesterol; HDL-C: High-density lipoprotein cholesterol; TG: triglycerides; BMI: Body Mass Index; BW: body weight.

**Table 4:** Isolation of lactobacilli from clinical cases of systemic infections.

**Patient’s age (year)/sex**	**Diagnosis**	**Underlying condition (s)**	**Organism (s) isolated**	**Therapy; duration of the therapy**	**Outcome**	**Ref.**
**46/M**	Bacteremia	Short-bowel syndrome, history of *Enterococcus faecalis* aortic valve endocarditis, *Klebsiella* pneumonia bacteremia & candidemia.	*L. confuses*; isolated from blood culture.	Piperacillin-tazobactam & gentamicin, 4 weeks.	Recovered.	[94]
**21/F**	Bacteremia	Fever, drownsiness & stiff neck.	*L. helvetica*; isolated from blood culture.	Amoxicillin & gentamicin, 10 days; clindamycin, 15 days.	Recovered	[95]
**59/F**	Bacteremia	Hypertension, diabetes mellitus type 2, history of breast cancer with conservative surgery & kidney stone.	*L. jensenii*; isolated from blood & urine culture.	Ampicillin, 2 weeks.	Recovered	[96]
**53/M**	Endocarditis	History of rheumatic fever.	*L. casei*; isolated from the blood & bone marrow.	Valve replacement surgery and was treated with doxycycline.	Recovered	[97]
**74/F**	Liver abscess	Hypertension, non-insulin-dependent diabetes mellitus & pneumonia.	*L. rhamnosus*; isolated from blood culture.	Ciprofloxacin & clindamycin, 6 weeks.	Recovered	[98]
